# Augmenting Standard Algorithms to Improve Dementia Detection and Predictive Accuracy in Medicare Beneficiaries

**DOI:** 10.1093/geroni/igaf122.4280

**Published:** 2025-12-31

**Authors:** Sara Knox, Janet Horn, Kit Simpson, Bethany Wolf

**Affiliations:** Medical University of South Carolina, Charleston, South Carolina, United States; Medical University of South Carolina, Medical University of South Carolina, South Carolina, United States; Medical University of South Carolina, Medical University of South Carolina, South Carolina, United States; Medical University of South Carolina, Medical University of South Carolina, South Carolina, United States

## Abstract

Accurate identification of Alzheimer’s Disease and Related Dementias (ADRD) in Medicare populations is critical for research and care planning. This study evaluated whether incorporating medication use and clinical diagnostic criteria into a digital phenotype could improve ADRD ascertainment over the standard CMS Chronic Conditions Warehouse (CCW) algorithm, which relies solely on diagnosis codes. Using data from 205,856 Medicare beneficiaries receiving home health services, we developed two predictive models: one based on the CCW phenotype and a novel ADRD (novel) phenotype based on clinical diagnostic criteria that categorizes individuals as ADRD-positive, ADRD-negative, or uncertain. Individuals with uncertain ADRD status were excluded during model training but analyzed later. Both models incorporated demographic variables, healthcare utilization, psychiatric symptoms, cognitive function, and prescription patterns. Data imputation and stacked elastic net regression were used to develop parsimonious models. Model performance was evaluated using AUC, sensitivity, specificity, and predictive values on an independent test set. The CCW model achieved an AUC of 0.863 for predicting its phenotype, while the novel model achieved an AUC of 0.906 for its respective phenotype. When applied to individuals with uncertain ADRD status, model predictions differed, with the novel model classifying more individuals as ADRD-negative than the CCW model. Overall, our findings suggest that augmenting standard claims-based algorithms with medication and clinical diagnostic criteria improves ADRD classification, particularly in cases where diagnosis is ambiguous. The novel model may offer a more conservative and specific approach to dementia identification, enhancing population-level research and targeted interventions.

